# 2216. Orthopedic Provider Adherence to American Academy of Orthopedic Surgeons (AAOS) Guidelines for Dental Prophylaxis in Total Joint Replacement

**DOI:** 10.1093/ofid/ofad500.1838

**Published:** 2023-11-27

**Authors:** Patricia Escaler, Thomas Myers, Katelyn Quartuccio, Sonal Munsiff, Louie Ted

**Affiliations:** University of Rochester Medical Center, DIvision of Infectious Diseases, Albany, New York; University of Rochester Medical Center, Rochester, New York; University of Rochester Medical Center, Highland Hospital, Rochester, NY; Univ. of Rochester, Rochester, NY; University of Rochester Medical Center, Rochester, New York

## Abstract

**Background:**

Antibiotic prophylaxis before dental procedures (DP) in patients with prosthetic joints has not been shown to decrease the risk of prosthetic joint infections (PJI). Current American Academy of Orthopedic Surgeons (AAOS) guidelines do not recommend DP in low-risk patients, and no longer mention clindamycin for penicillin allergic patients. The CDC recently prioritized antimicrobial stewardship (AS) for dental procedures. Orthopedic provider compliance with AAOS guidelines is unknown.

**Methods:**

This was a single-center, retrospective study of patients who underwent primary hip or knee replacement (TJR) from January to October 2019, and received DP prescribed by an orthopedic provider from the time of TJR through August 2021. DP was determined by chart review. Appropriateness was assessed according to 2016 AAOS guidelines. Further data was recorded, including demographics, pertinent orthopedic history, risk factors for PJI, and possible adverse effects from DP.

**Results:**

Of 1800 patients that were evaluated, 250 patients (13.9% of TJR) were given 258 prescriptions for DP. 64.8% were prescribed by advanced practice providers. Of the patients who received DP, 66.8% were female, and 22.5% were obese. There were slightly more hip replacements than knee replacements. 2.7% of patients were immunocompromised per AAOS definitions. Of the 33 diabetic patients, 87.9% were well-controlled, and thus considered low- risk. 240/258 prescriptions for DP (93%) were inappropriate, with the great majority of those in low-risk patients. Five patients inappropriately received clindamycin. Two patients received the incorrect dosage of antibiotic. Within 6 months of DP, C. difficile was diagnosed in 0.4% of patients, and resistant organisms from any source were cultured in 0.4% of patients. One patient developed PJI within one year of TJR, despite receiving DP.

**Conclusion:**

Though only a minority of patients undergoing TJR received DP, most were low-risk patients and thus considered inappropriate per 2016 AAOS guidelines. Further study is warranted, including analysis of dentists and other providers’ prescribing of DP, use of targeted educational materials, and utility of electronic health record order sets.

**Disclosures:**

**All Authors**: No reported disclosures

